# The mitochondrial genome of *Sinentomon erythranum *(Arthropoda: Hexapoda: Protura): an example of highly divergent evolution

**DOI:** 10.1186/1471-2148-11-246

**Published:** 2011-08-27

**Authors:** Wan-Jun Chen, Yun Bu, Antonio Carapelli, Romano Dallai, Sheng Li, Wen-Ying Yin, Yun-Xia Luan

**Affiliations:** 1 Key Laboratory of Insect Developmental and Evolutionary Biology, Institute of Plant Physiology and Ecology, Shanghai Institutes for Biological Sciences, Chinese Academy of Sciences, Shanghai 200032, China; 2 Department of Evolutionary Biology, University of Siena, I-53100 Siena, Italy

## Abstract

**Background:**

The phylogenetic position of the Protura, traditionally considered the most basal hexapod group, is disputed because it has many unique morphological characters compared with other hexapods. Although mitochondrial genome information has been used extensively in phylogenetic studies, such information is not available for the Protura. This has impeded phylogenetic studies on this taxon, as well as the evolution of the arthropod mitochondrial genome.

**Results:**

In this study, the mitochondrial genome of *Sinentomon erythranum *was sequenced, as the first proturan species to be reported. The genome contains a number of special features that differ from those of other hexapods and arthropods. As a very small arthropod mitochondrial genome, its 14,491 nucleotides encode 37 typical mitochondrial genes. Compared with other metazoan mtDNA, it has the most biased nucleotide composition with T = 52.4%, an extreme and reversed AT-skew of -0.351 and a GC-skew of 0.350. Two tandemly repeated regions occur in the A+T-rich region, and both could form stable stem-loop structures. Eighteen of the 22 tRNAs are greatly reduced in size with truncated secondary structures. The gene order is novel among available arthropod mitochondrial genomes. Rearrangements have involved in not only small tRNA genes, but also PCGs (protein-coding genes) and ribosome RNA genes. A large block of genes has experienced inversion and another nearby block has been reshuffled, which can be explained by the tandem duplication and random loss model. The most remarkable finding is that *trnL2(UUR) *is not located between *cox1 *and *cox2 *as observed in most hexapod and crustacean groups, but is between *rrnL *and *nad1 *as in the ancestral arthropod ground pattern. The "*cox1*-*cox2*" pattern was further confirmed in three more representative proturan species. The phylogenetic analyses based on the amino acid sequences of 13 mitochondrial PCGs suggest *S*. *erythranum *failed to group with other hexapod groups.

**Conclusions:**

The mitochondrial genome of *S. erythranum *shows many different features from other hexapod and arthropod mitochondrial genomes. It underwent highly divergent evolution. The "*cox1*-*cox2*" pattern probably represents the ancestral state for all proturan mitogenomes, and suggests a long evolutionary history for the Protura.

## Background

The Protura is a group of mysterious soil-dwelling micro-arthropods (usually 0.5-2.0 mm in length), first described by Silvestri in 1907 [1]. Traditionally, it was regarded as a basal hexapod group, but it owns many unique and primitive morphological characteristics compared with other hexapods. For example, they lack antennae and wings, the foretarsus are enlarged with many sensilla serving the role of antennae, eyes and tentorium are absent, they have anamorphic post-embryonic development, and they have 12 abdominal segments (instead of 11) [2]. The proturan spermatozoan has a variable number of doublet microtubules (9-16), with no accessory or central microtubules. It is different from those of other hexapods, but similar to the sperm of sea spider (Arthropoda: Pycnogonida). This probably reflects a high diversification rate, or a lengthy evolution [3-5]. Historically, there were many controversies about the relationship of proturans to other hexapods, and their evolutionary position in the Arthropoda [2,3,6-9]. This is because proturans are understudied, being so small and rare, making them difficult to collect, identify, culture and experiment on [2,10,11].

The higher-level phylogeny of the major arthropod groups (Chelicerata, Myriapoda, Crustacea and Hexapoda) continues to be a matter of debate despite extensive research based on phylogenetic analysis and genetic data [12-14]. Almost all molecular analyses strongly support the Pancrustacea hypothesis: crustaceans, instead of myriapods, are the closest relatives of the hexapods [15-18]. The Hexapoda (Insecta *s. lat*.), which includes four groups, Protura, Collembola, Diplura and Insecta (Insecta *s. str*.), was traditionally considered a monophyletic lineage based on the synapomorphies of body segments, six legs on the thorax, and adaptation to the terrestrial environment. The monophyly of the Insecta has been well established by morphological and molecular studies [8,10,17,18], but the monophyly of the Hexapoda is less certain [17,19]. Three basal hexapod groups (Protura, Collembola and Diplura) show many different features from insects according to morphology [10,20] and ultrastructure of spermatozoa [4]. The mitogenomic data of basal hexapod collembolans and diplurans reject the monophyly of Hexapoda, and suggest that some crustaceans are more closely related to the Insecta than Collembola and Diplura [17,19,21]. However, recent studies based on EST data and nuclear genes (18S and 28S ribosomal RNA genes, nuclear protein-coding sequences) support the monophyly of the Hexapoda [12,13,18].

The arthropod mitochondrial genome is a single circular DNA molecule encoding 13 proteins, 22 transfer RNAs (tRNAs), two ribosomal RNAs (rRNAs), and one A+T-rich region for the control of replication and transcription of the mtDNA. It is used extensively for studying phylogenetic relationships at various taxonomic levels. Unlike nuclear molecular markers, mtDNA is of maternal inheritance, and does not experience intermolecular genetic recombination. In addition, the mitochondrial gene order can provide additional phylogenetic information, since rearrangements appear to be generally rare events, and most mitochondrial gene arrangements often remain unchanged over a long evolutionary period [22]. Mitogenomic data also strongly support the Pancrustacea hypothesis [14,17,23], especially with the evidence of the gene order [16,24]. The gene *trnL2 *(UUR) is located between *rrnL *and *nad1 *in the ancestral arthropod ground pattern, but is translocated to the position between *cox1 *and *cox2 *in Pancrustacea [16]. It has been considered a distinctive synapomorphic character for crustaceans and hexapods. The mitochondrial genomes of basal hexapod Collembola [25] and Diplura [26] also agree with the "*cox1-trnL2-cox2*" pattern. So far, no mitochondrial genome information is available for the Protura. This has impeded comprehensive discussions on the evolution of the arthropod mitochondrial genome, and the validity of using mtDNA to study the phylogeny of the Hexapoda [27-29].

In this study, we sequenced the complete mitochondrial genome of *Sinentomon erythranum *(Protura: Sinentomata: Sinentomidae), to describe the molecular features of the proturan mitochondrial genome, to judge how these evolved, and to see if it has any phylogenetic information, which may help resolve the discrepancy on the monophyly of the Hexapoda between mitochondrial and nuclear DNA markers.

## Results and Discussion

### General description of the mitochondrial genome of *S. erythranum*

**Figure 1:**
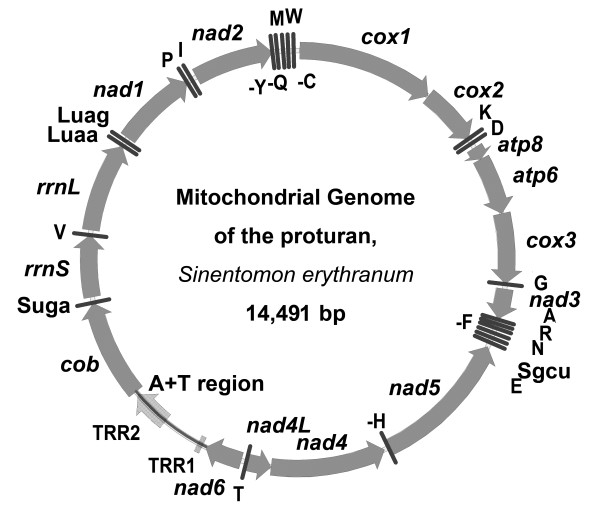
**Mitochondrial genome organization of *S. erythranum***. Protein-coding and ribosomal RNA genes are indicated with standard abbreviations, transfer RNA (tRNA) genes are designated by a single letter for the corresponding amino acid except for those coding for leucine and serine, which are labeled with their anticodon as well (Luag, Luaa, Sgcu and Suga). Arrows indicate direction of coding regions either on the J-strand (clockwise, 29 genes) or the N-strand (counterclockwise, eight genes) (after Simon 2006). The five tRNAs encoded by the N-strand are indicated by a (-) sign (for example -F). A+T region refers to the non-coding region that may be related to the regulation of mitochondrial replication and transcription. TRR stands for tandemly repeated region.

The mitochondrial genome of *S. erythranum *(GenBank accession HQ199311) encodes 37 genes, which is consistent with metazoan mitochondrial DNA structure (Figure 1 and Table 1). However, the total size of the genome is only 14,491 base pairs, smaller than most hexapod mitochondrial genomes, but similar in size to those of some spiders and mites (for example, the spider *Habronattus oregonensis *14381 bp, NC_005942). Most of the genes are encoded by the majority strand (J-strand, Simon et al. [30]), and only eight genes are encoded by the opposite strand (N-strand): five tRNAs and three protein-coding genes (PCGs) (*nad5*, *nad4*, *nad4L*). The gene order differs from that of the mitochondrial genomes of all sequenced arthropods, and most tRNA genes are reduced (Table 1). *trnW-uca *is the largest tRNA with 68 nucleotides, and the shortest tRNAs have only 53 nucleotides (*trnA-ugc*, *trnH-gug*, *trnV-uac*). The average size of all 22 tRNAs is less than 57 nucleotides. All 13 PCGs have the typical ATN start codon, and have either complete (TAA or TAG) or incomplete stop codons (TA (A), TA-, T--). The incomplete stop codons are presumably polyadenylated after transcription to form complete TAA stop codons [31]. The stop codons of several PCGs have an adenine (A) overlap with the next PCG's start codons. Such overlap is located at the junction of *cox1/cox2*, *atp8/atp6*, *atp6/cox3 *and *nad4L/nad4 *(Table 1).

**Table 1:** Annotation table for the mitochondrial genome of *S. erythranum*

Gene Name	Start	End	Strand	Start codon	Stop codon	Size(bp)	Intergenic(bp)
*cox1*	1	1532	+	ATG	TA(A)	1532	0
*cox2*	1533	2184	+	ATG	T--	652	0
*trnK-cuu*	2185	2246	+			62	-2
*trnD-uau*	2245	2299	+			55	1
*atp8*	2301	2446	+	ATG	TA(A)	146	0
*atp6*	2447	3093	+	ATA	TA(A)	647	0
*cox3*	3094	3876	+	ATG	TAA	783	0
*trnG-ucc*	3877	3931	+			55	0
*nad3*	3932	4270	+	ATT	TAA	339	8
*trnA-ugc*	4279	4331	+			53	-4
*trnR-ucg*	4328	4381	+			54	-7
*trnN-guu*	4375	4433	+			59	0
*trnF-gaa*	4434	4489	-			56	1
*trnS-gcu*	4491	4545	+			55	0
*trnE-uuc*	4546	4600	+			55	-1
*nad5*	4600	6198	-	ATA	TAA	1599	-4
*trnH-gug*	6195	6247	-			53	-1
*nad4*	6247	7528	-	ATA	TA-	1282	0
*nad4L*	7529	7806	-	ATG	TA(A)	278	2
*trnT-ugu*	7809	7862	+			54	5
*nad6*	7868	8287	+	ATT	TAG	420	993
*cob*	9281	10378	+	ATG	TAA	1098	4
*trnS-uga*	10383	10444	+			62	0
*rrnS*	10445	11134	+			690	0
*trnV-uac*	11135	11187	+			53	0
*rrnL*	11188	12183	+			996	12
*trnL-uaa*	12196	12250	+			55	4
*trnL-uag*	12255	12309	+			55	0
*nad1*	12310	13201	+	ATT	T--	892	0
*trnP-ugg*	13202	13256	+			55	5
*trnI-gau*	13262	13318	+			57	0
*nad2*	13319	14212	+	ATA	TAG	894	-3
*trnY-gua*	14210	14266	-			57	-2
*trnQ-uug*	14265	14330	-			66	-2
*trnM-cau*	14329	14384	+			56	2
*trnW-uca*	14387	14454	+			68	-18
*trnC-gca*	14437	14491	-			54	1

### Strand asymmetry

**Figure 2:**
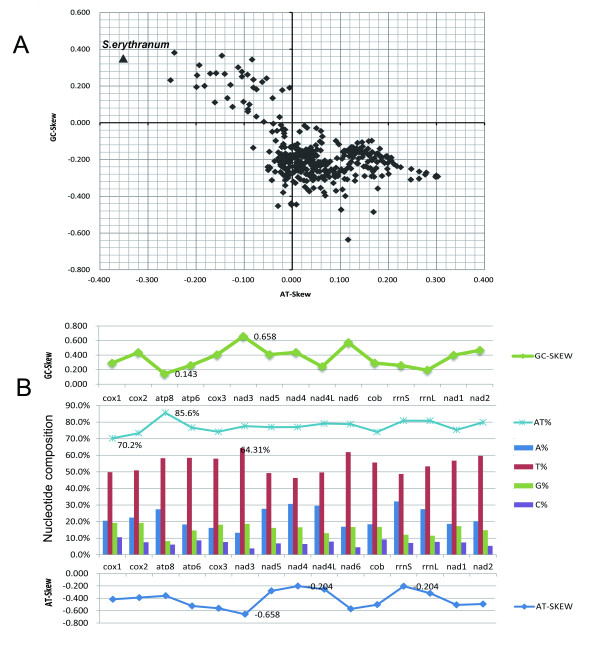
**Severe strand asymmetry of the mitochondrial genome sequence of *S. erythranum***. A. Scatterplots of skew values calculated for the whole majority strand for 360 arthropods. The value for *S. erythranum *(-0.351, 0.350) is indicated by the triangle at left, with the other arthropods represented by diamonds. B. Nucleotide composition (center), AT-skew (below) and GC-skew (above) of all 13 PCGs and two ribosome RNA genes of the *S. erythranum *mitogenome. All values are calculated for the majority strand.

Strand asymmetry (also called strand composition bias) is a remarkable feature of animal mitochondrial genomes. The overall mitogenomic AT-content of *S. erythranum *is 77.6%, which shows a strong bias towards A and T, and is well within the normal range of arthropod mtDNAs. The nucleotide frequency of the J-strand is T = 0.524, A = 0.252, G = 0.151, C = 0.073. Therefore, T is much more abundant than A, and G is more abundant than C. The AT-skew and GC-skew of the J-strand for *S. erythranum *are -0.351 and 0.350, respectively. They are extreme and reversed compared with those of most arthropods, which instead have a positive AT-skew and negative GC-skew (Figure 2A). The reversed value of AT-skew and GC-skew may indicate altered replication orientation of mtDNA in the A+T- rich region [32]. The skew value is the farthest of all from the coordinates (Figure 2A), meaning this proturan mitogenome has the most biased nucleotide composition ever reported for arthropods. The mitogenomic AT-skew value of *S. erythranum *(-0.351) is the most negative of all reported mitochondrial genomes, much lower than the second most-negative value from the American house dust mite *Dermatophagoides farinae *(NC_013184, AT-skew -0.253). For GC-skew, only the values of the small pigeon louse *Campanulotes bidentatus *(NC_007884, GC-skew 0.381) and tarantula *Calisoga longitarsis *(NC_010780, GC-skew 0.365) are slightly higher than the 0.350 of *S. erythranum*. It is unusual to find so many poly Ts within mitochondrial protein-coding sequences. For instance, a poly T motif in *cox3 *contains 27 continuous Ts, which results in the frequent use of TTT (F) codons. The exact reason for the occurrence of this motif remains unknown. In any case, the mitogenomic sequence of *S. erythranum *should be a good model for studying the mechanism of the base-frequency bias.

Figure 2B shows the nucleotide composition, AT-skew and GC-skew for each of the 13 PCGs and two rRNA genes of the mitochondrion of *S. erythranum*. *Cox1 *has the lowest AT content (70.2%) and *atp8 *has the highest AT content (85.6%). The AT content of these 15 genes does not fluctuate far from the overall average AT content (77.6%). *Nad3 *has the most negative AT-skew (-0.685), and *nad4 *and *rrnS *share the least extreme AT-skew (-0.204). The AT-skew values of the adjacent genes *nad5*, *nad4 *and *nad4L *are less extreme than in other adjacent genes, and all three of these genes are encoded by the minority strand, so it seems that some constraints shaped the genome that evolved under a strong directional mutation pressure (Figure 2B) [33].

### A+T-rich region

**Figure 3:**
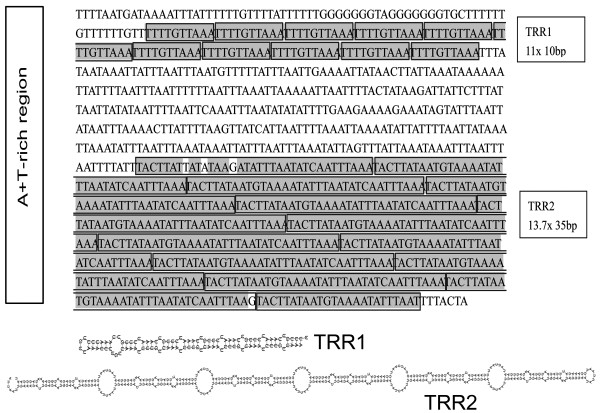
**Sequences of the A+T-rich region, primary and secondary structures of tandemly repeated regions (TRR): TRR1 (11 × 10 bp) and TRR2 (13.7 × 35 bp)**. In TRR2, the nucleotides that are not exactly same as the consensus pattern are shown in white background color.

The largest non-coding region (993 bp, Table 1), named the A+T-rich region in arthropods, is located between *nad6 *and *cob *(Figure 1), with a very high A+T content of 91.4% (Figure 3). There are two G-stretches (consisting of seven Gs each) at 5' of the A+T-rich region. The A+T-rich region contains two tandemly repeated regions (TRRs): TRR1 (11 × 10 bp) and TRR2 (13.7 × 35 bp). The repeat units are 'TTTTGTTAAA' for TRR1 and 'TACTTATAATGTAAAATATTTAATATCAATTTAAA' for TRR2. All 11 repeat units are exactly the same in TRR1, but for TRR2, only 11 repeat units are identical. Both TRRs can form stable stem-loop secondary structures (bottom of Figure 3). We noticed that the length of the A+T-rich region shows heteroplasmy at an intraspecific level [34]. Three kinds of length variations were detected by PCR amplification of the A+T-rich region from different individuals. The length heteroplasmy of the A+T-rich region is further confirmed by sequencing the PCR products after cloning. The copy number of TRR2 does vary in different individuals.

### Transfer RNAs

**Figure 4:**
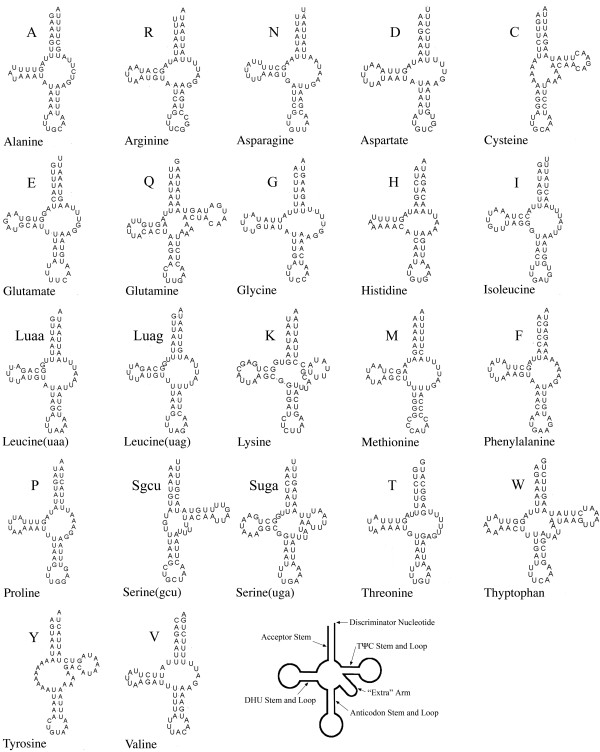
**Inferred tRNA secondary structures in the mitochondrial genome of *S. erythranum***.

The predicted secondary structures indicate that most tRNAs in our sequence have truncated structures (Figure 4). Among the 22 tRNAs, 15 of them lack a TΨC loop, and *trnS-gcu*, *trnY *and *trnC *lack the dihydrouridine (DHU) arm. The lack of the DHU arm in *trnS-gcu *is very common in metazoan mitochondrial genomes [35,36]. *trnC *is coded by the J-strand and shares 18 nucleotides with *trnW*, which is coded by the N-strand. Studies on nematode mtDNAs have proven that extremely reduced tRNAs, like those of *S. erythranum*, can function properly [37,38]. The extensive loss of the cloverleaf structures of tRNAs has been found in many groups of nematodes and arachnids [35,39,40], but to our knowledge, so many abnormal tRNA secondary structures within one mitochondrial genome have only been detected in very few hexapods, such as gall midges (Diptera: Cecidomyiidae) [41]. This suggests the independent origin of these truncated tRNA structural features in *S. erythranum *[38,41].

### Gene rearrangements and possible evolutionary mechanisms

**Figure 5:**
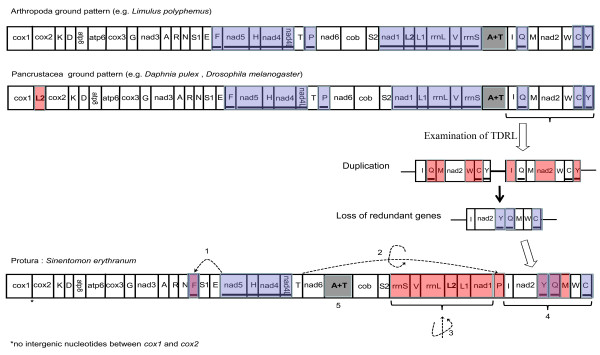
**Mitochondrial gene rearrangements in *S. erythranum *mtDNA compared with the ground patterns of Arthropoda and Pancrustacea, and the examination of the tandem duplication and random loss (TDRL) hypothesis**. Gene sizes are not drawn to scale. Genes encoded by the reverse strand are indicated by a dark line under the gene name with blue shadow. Red areas indicate genes that were rearranged, and circle arrows indicate inversion. The rearrangements are divided into five categories: 1) the translocation of *trnF*; 2) the remote translocation and inversion of *trnP*; 3) the local inversion of the gene block (*rrnS*, *trnV*, *rrnL*, *trnL2*, *trnL1*, and *nad1*); 4) the reshuffle of tRNAs region from *trnI *to *trnC*, which is compatible with the TDRL hypothesis; that is, duplication of the ancestral gene block from *trnI *to *trnY *can get the exact order of *S. erythranum*'s mtDNA in this region after loss of shadowed genes; 5) the relocation of the A+T-rich region.

Compared with the arthropod ground pattern (e.g. *Limulus polyphemus*), 11 of 37 genes in our proturan sequence have been rearranged: eight tRNA genes (*trnF*, *trnV*, *trnL2*, *trnL1*, *trnP*, *trnY*, *trnQ*, and *trnM*), two rRNA genes (*rrnS *and *rrnL*) and one PCG (*nad1*). The rearrangements can be divided into five categories (Figure 5): 1) the translocation of *trnF*; 2) the remote translocation and inversion of *trnP*; 3) the local inversion of the gene block (*rrnS*, *trnV*, *rrnL*, *trnL2*, *trnL1*, and *nad1*); 4) the reshuffle of the tRNA gene region from *trnI *to *trnC*; 5) the relocation of the A+T-rich region.

Rearrangements 1 and 2: the translocation of *trnF *may be an independent event, and this kind of minor rearrangement is very common in mtDNA [42,43]. The *trnP *changed its coding strand from N to J during its "long range" translocation, and this situation is rarely reported.

Rearrangements 3 and 4: The tandem duplication and random loss (TDRL) model is a popular hypothesis for explaining many mtDNA gene rearrangements [44-46]. Here, it can readily explain the reshuffling of tRNAs in the region from *trnI *to *trnC *(rearrangement 4 in Figure 5), although it does not explain the gene inversion (rearrangement 3 in Figure 5). For that inversion, the implication is strong that the gene block "*rrnS-V-rrnL-trnL2-trnL1-nad1*" was locally reversed as a whole. Gene inversions are probably the result of intra-molecular recombination, which can not only rearrange parts of the genome but also invert them at the same time. In the mitogenomic sequence of *S. erythranum*, both gene relocation and inversion must have occurred, although it is uncertain which of these two processes dominated. Here, we have some new thoughts. For the TDRL model, gene duplication is necessary, which can be achieved by replication slippage in single stranded templates. At the same time, a loop must be produced by slippage, so it is possible for the loop to perform intra-molecular recombination simultaneously [47]. Namely, the reshuffling of tRNAs and local inversion of a gene block may happen together in a stepwise rearrangement process. We further checked available mitochondrial genomes, and found that recombination involving PCGs has rarely occurred in hexapods, except in some lice whose mitochondrial genomes were extensively shuffled [48].

Rearrangement 5: it is not easy to explain the translocation of the A+T-rich region. There is a hint of an orientation change of replication due to the nucleotide-bias change from the majority type (AT-skew and GC-skew) (Figure 2A), but it is hard to explain it as a consequence of the inversion of gene block "*rrnS-V-rrnL-trnL2-trnL1-nad1*".

### Position of *trnL2*(UUR) and its phylogenetic implications

**Figure 6:**
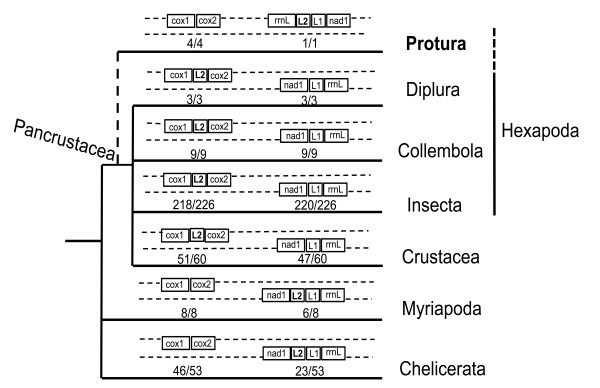
**Statistics and comparison of mitochondrial *trnL2 *patterns in all published mitochondrial genomes of arthropod lineages (until January 16, 2011)**. The ratios above the branches indicate the number of taxa with gene regions consistent with the pattern to the whole number of taxa whose mitogenomes are published.

The mitochondrial gene order of *S. erythranum *differs greatly from the pancrustacean ground pattern (Figure 5). The most remarkable finding is that *trnL2 *is not located between *cox1 *and *cox2*. The "*cox1-trnL2-cox2*" pattern was supposed to be a strong molecular evidence to support the Pancrustacea hypothesis [22]. *trnL2 *is located between *rrnL *and *nad1 *in the arthropod ground pattern, but is translocated to the position between *cox1 *and *cox2 *in crustaceans and hexapods. In our proturan sequence, *trnL2 *is found between *rrnL *and *nad1*, adjacent to *trnL1 *(*trnL-tag*). This is almost, but not quite, the arthropod ground pattern, that is, given the premise that the gene block "*rrnS-V-rrnL-trnL2-trnL1-nad1*" inverted as a whole, *trnL2 *and *trnL1 *must have changed their relative position compared with the arthropod ground pattern (Figures 5, 6). The gene sequences of *trnL2 *and *trnL1 *of *S. erythranum *are very similar (78% sequence identity, see detailed comparison between *trnL1 *and *trnL2 *in Additional File 1), so probably one *trnL *was copied from the other. This process can be explained by a mutational remolding hypothesis [49-51]. More mispairs appear in *trnL-uag *(*trnL1*) than in *trnL-uaa *(*trnL2*) (Additional File 1), so the *trnL-uaa *(*trnL2*) was most likely duplicated, and then one of the copies changed to *trnL-uag *by a random point-mutation of the anticodon triplet. After that, the original tRNA gene would have become a pseudogene or degenerated, so that the new *trnL-tag *replaced its function next to *trnL-taa*. In general, it cannot get a right paired tRNA duplicate from a wrong template, so we consider this as an evidence that *trnL2 *located between *rrnL *and *nad1 *is the ancestral state. Mitochondrial genomes of other basal hexapods (Diplura and Collembola) match the pancrustacean pattern of *cox1-trnL2-cox2 *[25,26]. Thus, the proturan *S. erythranum *is the only known hexapod whose *trnL2 *is in the ancestral arthropod position.

The Protura has three groups: Acerentomata, Sinentomata and Eosentomata. Besides *S. erythranum*, a member of the Sinentomata, we also sequenced the *cox1/cox2 *region (about 1.4 kb) from *Baculentulus tianmushanensis *of Acerentomata (GenBank accession HQ416715), *Eosentomon nivocolum *of Eosentomata (GenBank accession HQ416716), and *Zhongguohentomon piligeroum *of Eosentomata (GenBank accession HQ416714). They all agree with the *cox1-cox2 *pattern and have no intervening *trnL2*. In addition, *cox1 *is the exact neighbor to *cox2 *with no nucleotide between them in *S. erythranum*, *B. tianmushanensis *and *E. nivocolum*, and only four intergenic nucleotides in *Z. piligeroum*. Therefore, based on the available data, we believe it is more reasonable to conclude that the ancestral state is the *cox1-cox2 *pattern for all proturan mtDNAs.

The "*cox1-trnL2-cox2*" pattern occurs in almost all hexapods. We compared all published data of arthropod mitogenomes (available until January 16, 2011), and found only eight of 226 mtDNAs of Insecta are not consistent with this pattern (Figure 6 and Additional File 2), but they are clearly secondary mtDNA rearrangements or with multiple *trnL2 *copies. Five of them are from the Hemiptera, three parasitic lice from the Phthiraptera (*Bothriometopus macrocnemis*, *C. bidentatus compar *and *Heterodoxus macropus*) [52,53], one bark louse from the Psocoptera (*Lepidopsocid sp*. RS-2001) and one species from the Thysanoptera (*Thrips imaginis*) [54]. Their mitochondrial gene arrangements are reshuffled rigorously. The other three exceptions are from the Hymenoptera (*Vanhornia eucnemidarum*, *Abispa ephippium *and *Diadegma semiclausum*) [48]. It was noticed that in Hymenoptera, tRNA rearrangements (termed minor rearrangements) are very common, especially in the hot-spot areas [55]. In *Abispa ephippium*, *trnL2 *has four copies, but is still located between *cox1 *and *cox2 *[48]. However, most hemipteran and hymenopteran mtDNAs are still consistent with the *cox1-trnL2-cox2 *pattern. In Crustacea, only nine of 60 mitochondrial genomes are not consistent with the *cox1-trnL2-cox2 *pattern (Additional File 2). In addition, only seven of 53 mitochondrial genomes from the Chelicerata are not consistent with the *cox1*-*cox2 *pattern (Additional File 2), and all eight reported mitochondrial genomes from the Myriapoda are consistent with the *cox1*-*cox2 *pattern (Figure 6).

These statistics reflect the fact that translocation of *trnL2 *out of the *cox1/cox2 *junction has rarely happened within Pancrustacea lineage, and no case of the *cox1-trnL2-cox2 *pattern was detected within Myriapoda and Chelicerata lineages, whose *trnL2 *tends to stay between *rrnL *and *nad1*. This information leads to a single plausible scenario of the ancestral state being *cox1-trnL2-cox2 *in the Hexapoda, but the proturan mitochondrial genomes likely retain the ancestral state of the Arthropoda, the *cox1*-*cox2 *pattern. This seems to cast new doubt on the monophyly of Hexapoda. The Protura probably has a very ancient origin and a long evolutionary history, with distant affinity to other hexapods, evolving even earlier than other pancrustaceans. However, we cannot exclude the possibility of the secondary reversion to the primitive arthropod condition in the proturan ancestor since our gene sequence is so highly divergent. In this case, the mtDNA of *S. erythranum *provides a remarkable example of secondary reversion.

### Phylogenetic position of Protura

Since the position of *trnL2 *cast doubt on the relationship between the Protura and other hexapods, it is important to verify it with a phylogenetic tree. As revealed in Figure 2A, the base composition of *S. erythranum *is so different from that of most arthropod mitochondrial genomes, long-branch attraction (LBA) can be expected. Translating the PCGs into amino acid sequences is an effective method of dealing with the problem caused by base compositional heterogeneity in tree reconstruction [14,17,56], so we performed all phylogenetic analyses on conceptually translated amino acid data of 13 mitochondrial PCGs using maximum likelihood and Bayesian inference methods.

**Figure 7:**
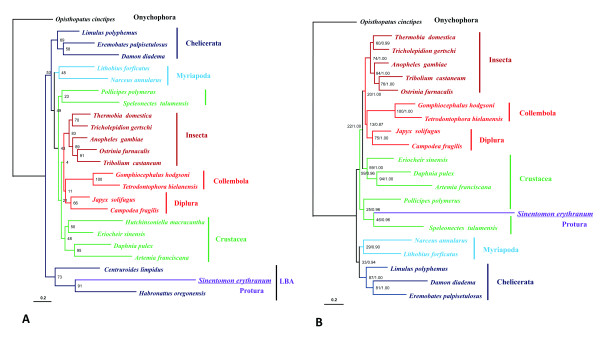
**Maximum likelihood trees of *S. erythranum *and other arthropod representatives based on the amino acid sequences of 13 mitochondrial PCGs**. A. 24 taxa. Numbers at each node indicate bootstrap values of maximum likelihood analysis (100 replicates). B. 21 taxa. Numbers at each node indicate bootstrap values of maximum likelihood analysis (100 replicates, BS) and Bayesian posterior probabilities (PP) in format of "BS/PP".

In the ML and Bayesian trees, *S. erythranum *displayed a remarkable long-branch, and clustered with other long-branches (Figure 7A). The AT-skew and GC-skew plot reveals that *Hutchinsoniella macracantha*, *Habronattus oregonensis *and *Centruroides limpidus *have a similar base composition to *S. erythranum *(negative AT-skew and positive GC-skew). After removing these three taxa, *S. erythranum *clustered with *Speleonectes tulumensis *(Crustacea: Remipedia), but the bootstrap value and posterior probability are relatively low, which prevent us from determining the exact phylogenetic position of the Protura (Figure 7B). We also tested the phylogenetic placement of *S. erythranum *by sequential taxon removal, and it consistently showed a distant affinity to the Insecta (data not shown).

In our trees (Figure 7), the clade of Diplura and Collembola is sister to Insecta, although the bootstrap value is relatively low. It is different from previous studies based on mitochondrial gene sequences of diplurans and collembolans, which suggested that some crustaceans are more closely related to Insecta than Collembola and Diplura [17]. More arthropod taxa are needed to further discuss this problem.

The unusual long-branch length indicates that the *S. erythranum *mitochondrial genomes are evolving rapidly. The population of soil-dwelling proturans is usually very small. Mutations may accumulate faster in such organisms due to the slow rate of gene flow. This also seems true for nematodes, parasitic lice and mites, in which high levels of genome diversity are commonly detected. The study on the mitochondrial genome of two diplurans also reveals that high genetic divergence existed in the morphologically uniform taxa [26].

Whether the Protura is a real hexapod group or not has been debated for a long time [7]. The Protura have many unique morphological characters compared with other hexapods: 1) they have no eyes and no antennae; 2) they have abdominal legs on abdominal segments 1-3; 3) they have no caudal cerci but have a telson tail, which is common in crustaceans but absent in other hexapods [1-3]; 4) the axoneme of flagellated spermatozoa lacks central microtubules, which is similar to the condition in pycnogonid spermatozoa [4]; 5) the serosa (embryonic membrane) of proturans retains the ability to differentiate into a tergum or definitive dorsal closure during embryonic development, which is similar to crustaceans and myriapods, but different from other hexapods. Based on information from embryonic development, Machida (2006) proposed that the Protura may have a much longer evolutionary history than previously thought [9]. However, a few recent studies based on EST data and rRNA genes have presented relatively robust evidence supporting the monophyly of Hexapoda and Pancrustacea (although only one proturan species was included in these studies) [12,18].

Although the mitochondrial genome sequence of *S*. *erythranum *is unique, with little phylogenetic affinity to the insects, we cannot equate this to the evolutionary history of the Protura. Mitochondrial genome data alone are not enough to unambiguously resolve the relationships of Protura, Diplura, Collembola and Insecta. It is necessary to understand the limits and applicability of these data [27]. Our sequence data showed many unique molecular features, which can provide valuable information for studying problems of mitochondrial genome evolution, for example, the mechanisms of mitochondrial gene rearrangements, truncation of tRNA secondary structures, and nucleotide frequency bias. Understanding these fundamental biology problems should be helpful in phylogenetic analyses when using mitochondrial genomic data.

## Conclusions

This is the first report of a complete mitochondrial genome from the Protura. With highly divergent evolution, their mtDNA has many different features to that of other hexapods, including nucleotide-frequency bias, gene order, and tRNA secondary structure. Therefore, it is a valuable example to study the mechanism of mitochondrial gene evolution and rearrangement in the Arthropoda.

Our study suggests that proturan mtDNAs do not agree with the "*cox1-trnL2-cox2*" pattern, which was thought to be an important character shared by hexapod and crustacean groups. It may be a result of secondary reversion due to extensive rapid and divergent evolution, but also may suggests that the Protura have a long evolutionary history, and do not have a close affinity to hexapods and crustaceans. *S. erythranum *did not group with other hexapods in our phylogenetic trees, and its extreme long-branch implies that its mtDNA underwent highly divergent evolution. More evidence is needed to verify this hypothesis and to solve the conflict between the studies on mitochondrial and nuclear gene markers.

## Methods

### mtDNA sequencing of *S. erythranum*

Specimens of *S. erythranum *were collected from Tianping Mountain (Jiangsu Province, China). The total DNA of one individual was extracted with the commercial kit Wizard SV Genomic Purification System (Promega), and then was used as the template for PCR amplifications. Initially, two small fragments of *cox1 *and *cob *were amplified using two universal primer pairs of LCO1490/HCO2198 [57] and CobF424/CobR876 [58], respectively, and the PCR products were sequenced directly by the amplification primers. Four primers were designed according to these obtained sequences for two long PCR amplifications encompassing the *cox1/cob *(~9 kb) and *cob/cox1 *(~6 kb) fragments, respectively. These primers were SI-C1-J320 (CTGGTTGAACTGTTTATCCTCCTC)/SI-Cb-N239 (ATAAGGATGAAAACTAACCCTATCA), and SI-Cb-J181 (GTTCTTCTAATCCTTTAGGAGTTGG)/SI-C1-N343 (GAGGAGGATAAACAGTTCAACCAG). Long PCRs were generated with LA Taq (Takara, Dalian, China) under the following two-step conditions: 35 cycles of 96°C for 2 min and 68°C for 10 min, followed by incubation at 68°C for 10 min. The 9 kb and 6 kb products were mixed together after gel-purification, and then sequenced with the shotgun sequencing approach as described by Masta and Boore (2004) [39]. The sequencing service was from Shanghai Majorbio Biotech Co., Ltd. Two contigs were assembled by Phred/Phrap [59,60] from the shotgun sequencing readings, guaranteed to have 10 times coverage for both contigs. More specific primers were designed for PCR amplifications to bridge two remaining gaps (primers available on request). All PCR products were then cloned and then sequenced by an ABI 3730 automated DNA sequencer. A consensus sequence was assembled from all the contigs using Seqman in the DNAStar software package (DNASTAR Inc., Madison, WI) [61].

### Gene annotation and secondary structure prediction

The sequence was submitted in Fasta format to the web-based software DOGMA (Dual Organellar Genome Annotator) [62] for primary annotation. BLAST searches were done on NCBI Blast Entrez databases to ensure the identity of PCGs and rRNA genes. To identify the tRNA genes in the genome, we used the annotation obtained by DOGMA (with the COVE threshold for tRNAs set to 7(low)), and further used tRNAscan-SE via the web interface and the "Nematode Mito" settings for the COVE program [63]. The ARWEN (version 1.2) program was also used by the web interface with the "mtmam" option switched off [64]. Finally, the tRNAs were determined by comparing the secondary structures suggested by these different programs. Tandemly repetitive sequences in the A+T-rich region were determined both manually and by using the Tandem Repeats Finder [65]. The putative minimum-free-energy structures of TRRs were given by RNAfold WebServer in the Vienna RNA Websuite [66].

### Sequence determination of *cox1/cox2 *junction region

In order to find if *trnL2 *lay outside of *cox1 *and *cox2*, not only in the Sinentomata but also in the other proturan groups, we amplified and sequenced the *cox1/cox2 *junction (about 1.4 kb) of *B. tianmushanensis *(Acerentomata: Berberentomidae), *E. nivocolum *(Eosentomata: Eosentomidae) and *Z. piligeroum *(Eosentomata: Eosentomidae) using the universal primer pair C1-HCO-J and C2-B-3665 [30]. We followed the above-mentioned methods to annotate these genes.

### Statistical comparison of strand asymmetry and of *trnL2 *positions of arthropod mtDNAs

We retrieved the nucleotide sequences and DNA compositions for all 359 published arthropod mtDNAs (before January 16, 2011) from the Mitome database [67] or NCBI Organelle Genome Resources. Strand asymmetry represents strand compositional bias, usually reflected by the AT skew = (A-T)/(A+T) and GC-skew = (G-C)/(G+C) [32,68].

We further checked the position of *trnL2 *in all 359 available arthropod mtDNAs. For the pancrustacean groups, we checked whether each mtDNA agreed with the typical patterns of *cox1-trnL2-cox2 *and *rrnL-trnL1-nad1*; then, we did the same for the other arthropods, the myriapods and chelicerates, which typically have the different pattern of *cox1-cox2 *and *rrnL-trnL1-trnL2-nad1 *[16].

### Phylogenetic Analysis

First, we choose 24 Panarthropoda representatives (Additional File 3) for phylogenetic tree construction based on previous studies [14,17], including three groups with the similar base composition to *S. erythranum *(negative AT-skew and positive GC-skew, Additional File 4), in order to see if *S. erythranum *will group with them because of LBA. Then, we reconstructed the phylogenetic trees after removing these three taxa, focusing on the relationship of *S. erythranum *and other hexapods. The onychophoran *Opisthopatus cinctipes *was defined as the outgroup in our analyses.

The nucleotide sequences of each PCG were retro-aligned using DAMBE, version 5.1.1 [69]. The 13 amino acid data were concatenated as an alignment of 3819 positions after individually aligned, and then, 2520 aligned characters for 24 taxa and 2616 aligned characters for 21 taxa were retained respectively after Gblocks screening with default settings [70]. The best model "mtREV24+G+I+F" was selected using MEGA 5.0 [71]. We carried out ML searches with RAxML through the web portal http://phylobench.vital-it.ch/raxml-bb/index.php[72]. Bayesian analysis was performed using MrBayes (version 3.1.2), with mtRev+I+G model [73]. Four Markov chains were run for 1,000,000 generations, and sampled every 100 generations to yield a posterior probability distribution of 10,000 trees. The first 2,000 trees were discarded as burn-in. The standard deviation of split frequencies was lower than 0.01 in 21 taxa dataset analysis, but we failed to obtain a meaningful convergence for the 24 taxa dataset.

## Authors' contributions

WJC carried out most of the experimental work, performed the molecular analyses and drafted the manuscript. YB sampled the specimens, participated in the molecular experiment and data analyses. AC analyzed the data and drew tRNA structures. RD, SL, WYY provided intellectual contributions during the implementation of this study. YXL supervised the study, analyzed the data and wrote the manuscript. All authors read and approved the final manuscript.

## Supplementary Material

Additional File 1 **The comparison of gene sequences and secondary structures between *trnL1*-uag and *trnL2*-uaa**. Click here for file

Additional File 2 **List of 24 mitochondrial genomes, which are not compatible with the "*cox1-trnL2-cox2*" pattern from Insecta and Crustacea, and not consistent with the "*cox1-cox2*" pattern from Chelicerata**. Click here for file

Additional File 3 **List of 24 taxa used in the phylogenetic analysis and the base composition of their mitochondrial genomes**. Click here for file

Additional File 4 **AT-skew and GC-skew plot for 24 taxa used in phylogenetic analysis**. Click here for file

## References

[B1] Silvestri F Descrizione di un novo genere d'insetti apterigoti rappresentante di un novo ordine Boll Lab Zool Portici 1907 1 296 311

[B2] Yin WY Fauna Sinica Arthropoda: Protura 1999 Beijing: Science Press in Chinese with English summary

[B3] Yin WY A new idea on phylogeny of Protura with approach to its origin and systematic position Sci Sin Ser B 1984 27 149 160

[B4] Yin WY Xue LZ Comparative Spermatology of Protura and Its Significance on Proturan Systematics Sci China Ser B 1993 36 5 575 586

[B5] Dallai R Mercati D Bu Y Yin YW Callaini G Riparbelli MG The spermatogenesis and sperm structure of *Acerentomon microrhinus *(Protura, Hexapoda) with considerations on the phylogenetic position of the taxon Zoomorphology 2010 129 1 61 80 10.1007/s00435-009-0100-1

[B6] Hennig W Insect phylogeny 1981 New York: John Wiley & Sons

[B7] Dallai R Simonetta AM, Morris SC Are Protura really insects? The Early Evolution of Metazoa and the Significance of Problematic Taxa 1991 Cambridge: The Cambridge University Press 263 269

[B8] Luan YX Mallatt JM Xie RD Yang YM Yin WY The phylogenetic positions of three basal-hexapod groups (Protura, Diplura, and Collembola) based on ribosomal RNA gene sequences Mol Biol Evol 2005 22 7 1579 1592 10.1093/molbev/msi148 15845456

[B9] Machida R Evidence from embryology for reconstructing the relationships of hexapod basal clades Arthropod Systematics & Phylogeny 2006 64 1 95 10421830722

[B10] Bitsch C Bitsch J Phylogenetic relationships of basal hexapods among the mandibulate arthropods: a cladistic analysis based on comparative morphological characters Zoolog Sci 2004 33 6 511 550 10.1111/j.0300-3256.2004.00162.x

[B11] Szeptycki A Catalogue of the world Protura Acta Zoologica Cracoviensia 2007 50B 1

[B12] Meusemann K von Reumont BM Simon S Roeding F Strauss S Kuck P Ebersberger I Walzl M Pass G Breuers S A phylogenomic approach to resolve the arthropod tree of life Mol Biol Evol 2010 27 11 2451 2464 10.1093/molbev/msq130 20534705

[B13] Regier JC Shultz JW Zwick A Hussey A Ball B Wetzer R Martin JW Cunningham CW Arthropod relationships revealed by phylogenomic analysis of nuclear protein-coding sequences Nature 2010 463 7284 1079 1083 10.1038/nature08742 20147900

[B14] Rota-Stabelli O Kayal E Gleeson D Daub J Boore JL Telford MJ Pisani D Blaxter M Lavrov DV Ecdysozoan mitogenomics: evidence for a common origin of the legged invertebrates, the Panarthropoda Genome Biol Evol 2010 2 425 440 10.1093/gbe/evq030 20624745 PMC2998192

[B15] Friedrich M Tautz D Ribosomal DNA phylogeny of the major extant arthropod classes and the evolution of myriapods Nature 1995 376 6536 165 167 10.1038/376165a0 7603566

[B16] Boore JL Lavrov DV Brown WM Gene translocation links insects and crustaceans Nature 1998 392 6677 667 668 10.1038/33577 9565028

[B17] Carapelli A Lio P Nardi F van der Wath E Frati F Phylogenetic analysis of mitochondrial protein coding genes confirms the reciprocal paraphyly of Hexapoda and Crustacea BMC Evol Biol 2007 7 Suppl 2 S8 10.1186/1471-2148-7-S2-S8 17767736 PMC1963475

[B18] Mallatt J Craig CW Yoder MJ Nearly complete rRNA genes assembled from across the metazoan animals: Effects of more taxa, a structure-based alignment, and paired-sites evolutionary models on phylogeny reconstruction Mol Phylogenet Evol 2010 55 1 1 17 10.1016/j.ympev.2009.09.028 19786108

[B19] Nardi F Spinsanti G Boore JL Carapelli A Dallai R Frati F Hexapod origins: monophyletic or paraphyletic? Science 2003 299 5614 1887 1889 10.1126/science.1078607 12649480

[B20] Manton SM Harding MJP The evolution of arthropodan locomotory mechanisms. Part 10. Locomotory habits, morphology and evolution of the hexapod classes Zool J Linn Soc-Lond 1972 51 203 400 10.1111/j.1096-3642.1972.tb02550.x

[B21] Cook CE Yue QY Akam M Mitochondrial genomes suggest that hexapods and crustaceans are mutually paraphyletic P Roy Soc B-Biol Sci 2005 272 1569 1295 1304 10.1098/rspb.2004.3042 PMC1564108 16024395

[B22] Boore JL Animal mitochondrial genomes Nucleic Acids Res 1999 27 8 1767 1780 10.1093/nar/27.8.1767 10101183 PMC148383

[B23] Negrisolo E Minelli A Valle G The mitochondrial genome of the house centipede scutigera and the monophyly versus paraphyly of myriapods Mol Biol Evol 2004 21 4 770 780 10.1093/molbev/msh078 14963096

[B24] Boore JL Collins TM Stanton D Daehler LL Brown WM Deducing the pattern of arthropod phylogeny from mitochondrial DNA rearrangements Nature 1995 376 6536 163 165 10.1038/376163a0 7603565

[B25] Nardi F Carapelli A Fanciulli PP Dallai R Frati F The complete mitochondrial DNA sequence of the basal hexapod Tetrodontophora bielanensis: evidence for heteroplasmy and tRNA translocations Mol Biol Evol 2001 18 7 1293 1304 11420368 10.1093/oxfordjournals.molbev.a003914

[B26] Podsiadlowski L Carapelli A Nardi F Dallai R Koch M Boore JL Frati F The mitochondrial genomes of *Campodea fragilis *and *Campodea lubbocki *(Hexapoda: Diplura): High genetic divergence in a morphologically uniform taxon Gene 2006 381 49 61 16919404 10.1016/j.gene.2006.06.009

[B27] Cameron SL Miller KB D'Haese CA Whiting MF Barker SC Mitochondrial genome data alone are not enough to unambiguously resolve the relationships of Entognatha, Insecta and Crustacea sensu lato (Arthropoda) Cladistics 2004 20 6 534 557 10.1111/j.1096-0031.2004.00040.x 34892962

[B28] Glenner H Thomsen PF Hebsgaard MB Sorensen MV Willerslev E Evolution. The origin of insects Science 2006 314 5807 1883 1884 10.1126/science.1129844 17185588

[B29] Budd GE Telford MJ The origin and evolution of arthropods Nature 2009 457 7231 812 817 10.1038/nature07890 19212398

[B30] Simon C Buckley TR Frati F Stewart JB Beckenbach AT Incorporating molecular evolution into phylogenetic analysis, and a new compilation of conserved polymerase chain reaction primers for animal mitochondrial DNA Annu Rev Ecol Evol S 2006 37 545 579 10.1146/annurev.ecolsys.37.091305.110018

[B31] Ojala D Montoya J Attardi G tRNA punctuation model of RNA processing in human mitochondria Nature 1981 290 5806 470 474 10.1038/290470a0 7219536

[B32] Wei SJ Shi M Chen XX Sharkey MJ van Achterberg C Ye GY He JH New Views on Strand Asymmetry in Insect Mitochondrial Genomes Plos One 2010 5 9 10.1371/journal.pone.0012708 PMC2939890 20856815

[B33] Hassanin A Leger N Deutsch J Evidence for multiple reversals of asymmetric mutational constraints during the evolution of the mitochondrial genome of metazoa, and consequences for phylogenetic inferences Syst Biol 2005 54 2 277 298 10.1080/10635150590947843 16021696

[B34] Zhang DX Hewitt GM Insect mitochondrial control region: A review of its structure, evolution and usefulness in evolutionary studies Biochem Syst Ecol 1997 25 2 99 120 10.1016/S0305-1978(96)00042-7

[B35] Wolstenholme DR Macfarlane JL Okimoto R Clary DO Wahleithner JA Bizarre tRNAs inferred from DNA sequences of mitochondrial genomes of nematode worms Proc Natl Acad Sci USA 1987 84 5 1324 1328 10.1073/pnas.84.5.1324 3469671 PMC304420

[B36] Garey JR Wolstenholme DR Platyhelminth mitochondrial DNA: evidence for early evolutionary origin of a tRNA(serAGN) that contains a dihydrouridine arm replacement loop, and of serine-specifying AGA and AGG codons J Mol Evol 1989 28 5 374 387 10.1007/BF02603072 2545889

[B37] Lavrov DV Brown WM Boore JL A novel type of RNA editing occurs in the mitochondrial tRNAs of the centipede *Lithobius forficatus* Proc Natl Acad Sci USA 2000 97 25 13738 13742 10.1073/pnas.250402997 11095730 PMC17645

[B38] Segovia R Pett W Trewick S Lavrov DV Extensive and evolutionarily persistent mitochondrial tRNA editing in velvet worms (phylum Onychophora) Mol Biol Evol 2011 10.1093/molbev/msr113 21546355

[B39] Masta SE Boore JL The complete mitochondrial genome sequence of the spider *Habronattus oregonensis *reveals rearranged and extremely truncated tRNAs Mol Biol Evol 2004 21 5 893 902 10.1093/molbev/msh096 15014167

[B40] Klimov PB OConnor BM Improved tRNA prediction in the American house dust mite reveals widespread occurrence of extremely short minimal tRNAs in acariform mites Bmc Genomics 2009 10 598 10.1186/1471-2164-10-598 20003349 PMC2797822

[B41] Beckenbach AT Joy JB Evolution of the mitochondrial genomes of gall midges (Diptera: Cecidomyiidae): rearrangement and severe truncation of tRNA genes Genome Biol Evol 2009 1 278 287 20333197 10.1093/gbe/evp027 PMC2817422

[B42] Moritz C Dowling TE Brown WM Evolution of Animal Mitochondrial-DNA - Relevance for Population Biology and Systematics Annu Rev Ecol Syst 1987 18 269 292 10.1146/annurev.es.18.110187.001413

[B43] Negrisolo E Minelli A Valle G Extensive gene order rearrangement in the mitochondrial genome of the centipede *Scutigera coleoptrata* J Mol Evol 2004 58 4 413 423 10.1007/s00239-003-2563-x 15114420

[B44] Macey JR Larson A Ananjeva NB Fang ZL Papenfuss TJ Two novel gene orders and the role of light-strand replication in rearrangement of the vertebrate mitochondrial genome Mol Biol Evol 1997 14 1 91 104 9000757 10.1093/oxfordjournals.molbev.a025706

[B45] Lavrov DV Boore JL Brown WM Complete mtDNA sequences of two millipedes suggest a new model for mitochondrial gene rearrangements: Duplication and nonrandom loss Mol Biol Evol 2002 19 2 163 169 11801744 10.1093/oxfordjournals.molbev.a004068

[B46] San Mauro D Gower DJ Zardoya R Wilkinson M A hotspot of gene order rearrangement by tandem duplication and random loss in the vertebrate mitochondrial genome Mol Biol Evol 2006 23 1 227 234 16177229 10.1093/molbev/msj025

[B47] Lunt DH Hyman BC Animal mitochondrial DNA recombination Nature 1997 387 6630 247 247 10.1038/387247a0 9153388

[B48] Cameron SL Dowton M Castro LR Ruberu K Whiting MF Austin AD Diement K Stevens J Mitochondrial genome organization and phylogeny of two vespid wasps Genome 2008 51 10 800 808 10.1139/G08-066 18923531

[B49] Rawlings TA Collins TM Bieler R Changing identities: tRNA duplication and remolding within animal mitochondrial genomes Proc Natl Acad Sci USA 2003 100 26 15700 15705 10.1073/pnas.2535036100 14673095 PMC307631

[B50] Covacin C Shao R Cameron S Barker SC Extraordinary number of gene rearrangements in the mitochondrial genomes of lice (Phthiraptera: Insecta) Insect Mol Biol 2006 15 1 63 68 10.1111/j.1365-2583.2005.00608.x 16469069

[B51] Kilpert F Podsiadlowski L The Australian fresh water isopod (Phreatoicidea: Isopoda) allows insights into the early mitogenomic evolution of isopods Comp Biochem Phys D 2010 5 1 36 44 10.1016/j.cbd.2009.09.003 20374940

[B52] Shao RF Barker SC The highly rearranged mitochondrial genome of the plague thrips, Thrips imaginis (Insecta: thysanoptera): Convergence of two novel gene boundaries and an extraordinary arrangement of rRNA genes Mol Biol Evol 2003 20 3 362 370 10.1093/molbev/msg045 12644556

[B53] Shao R Dowton M Murrell A Barker SC Rates of gene rearrangement and nucleotide substitution are correlated in the mitochondrial genomes of insects Mol Biol Evol 2003 20 10 1612 1619 10.1093/molbev/msg176 12832626

[B54] Shao R Campbell NJ Schmidt ER Barker SC Increased rate of gene rearrangement in the mitochondrial genomes of three orders of hemipteroid insects Mol Biol Evol 2001 18 9 1828 1832 11504862 10.1093/oxfordjournals.molbev.a003970

[B55] Dowton M Castro LR Campbell SL Bargon SD Austin AD Frequent mitochondrial gene rearrangements at the hymenopteran nad3-nad5 junction J Mol Evol 2003 56 5 517 526 10.1007/s00239-002-2420-3 12698290

[B56] Sheffield NC Song H Cameron SL Whiting MF Nonstationary evolution and compositional heterogeneity in beetle mitochondrial phylogenomics Syst Biol 2009 58 4 381 394 10.1093/sysbio/syp037 20525592

[B57] Folmer O Black M Hoeh W Lutz R Vrijenhoek R DNA primers for amplification of mitochondrial cytochrome c oxidase subunit I from diverse metazoan invertebrates Mol Mar Biol Biotechnol 1994 3 5 294 299 7881515

[B58] Boore JL Brown WM Mitochondrial genomes of Galathealinum, Helobdella, and Platynereis: sequence and gene arrangement comparisons indicate that Pogonophora is not a phylum and Annelida and Arthropoda are not sister taxa Mol Biol Evol 2000 17 1 87 106 10666709 10.1093/oxfordjournals.molbev.a026241

[B59] Ewing B Hillier L Wendl MC Green P Base-calling of automated sequencer traces using phred. I. Accuracy assessment Genome Res 1998 8 3 175 185 9521921 10.1101/gr.8.3.175

[B60] Ewing B Green P Base-calling of automated sequencer traces using phred. II. Error probabilities Genome Res 1998 8 3 186 194 9521922

[B61] Burland TG DNASTAR's Lasergene sequence analysis software Methods Mol Biol 2000 132 71 91 10547832 10.1385/1-59259-192-2:71

[B62] Wyman SK Jansen RK Boore JL Automatic annotation of organellar genomes with DOGMA Bioinformatics 2004 20 17 3252 3255 10.1093/bioinformatics/bth352 15180927

[B63] Lowe TM Eddy SR tRNAscan-SE: A program for improved detection of transfer RNA genes in genomic sequence Nucleic Acids Res 1997 25 5 955 964 10.1093/nar/25.5.955 9023104 PMC146525

[B64] Laslett D Canback B ARWEN: a program to detect tRNA genes in metazoan mitochondrial nucleotide sequences Bioinformatics 2008 24 2 172 175 10.1093/bioinformatics/btm573 18033792

[B65] Benson G Tandem repeats finder: a program to analyze DNA sequences Nucleic Acids Res 1999 27 2 573 580 10.1093/nar/27.2.573 9862982 PMC148217

[B66] Gruber AR Lorenz R Bernhart SH Neubock R Hofacker IL The Vienna RNA websuite Nucleic Acids Res 2008 36 Web Server W70 74 10.1093/nar/gkn188 18424795 PMC2447809

[B67] Lee YS Oh J Kim YU Kim N Yang S Hwang UW Mitome: dynamic and interactive database for comparative mitochondrial genomics in metazoan animals Nucleic Acids Res 2008 36 D938 D942 17940090 10.1093/nar/gkm763 PMC2238945

[B68] Perna NT Kocher TD Patterns of Nucleotide Composition at Fourfold Degenerate Sites of Animal Mitochondrial Genomes J Mol Evol 1995 41 3 353 358 10.1007/BF01215182 7563121

[B69] Xia X Xie Z DAMBE: software package for data analysis in molecular biology and evolution J Hered 2001 92 4 371 373 10.1093/jhered/92.4.371 11535656

[B70] Castresana J Selection of conserved blocks from multiple alignments for their use in phylogenetic analysis Mol Biol Evol 2000 17 4 540 552 10742046 10.1093/oxfordjournals.molbev.a026334

[B71] Tamura K Peterson D Peterson N Stecher G Nei M Kumar S MEGA5: Molecular Evolutionary Genetics Analysis using Maximum Likelihood, Evolutionary Distance, and Maximum Parsimony Methods Mol Biol Evol in press 10.1093/molbev/msr121 PMC3203626 21546353

[B72] Stamatakis A Hoover P Rougemont J A Rapid Bootstrap Algorithm for the RAxML Web Servers Syst Biol 2008 57 5 758 771 10.1080/10635150802429642 18853362

[B73] Huelsenbeck JP Ronquist F MRBAYES: Bayesian inference of phylogenetic trees Bioinformatics 2001 17 8 754 755 10.1093/bioinformatics/17.8.754 11524383

