# Supplementary material for: The mitochondrial genome of Sinentomon erythranum (Arthropoda: Hexapoda: Protura): an example of highly divergent evolution
Source: BMC Evol Biol. 2011 Aug 27;11:246. doi: 10.1186/1471-2148-11-246 (PMC3176236; doi:10.1186/1471-2148-11-246)
Supplement: Additional File 1 — The comparison of gene sequences and secondary structures between trnL1-uag and trnL2-uaa. [file 1471-2148-11-246-S1.PDF]

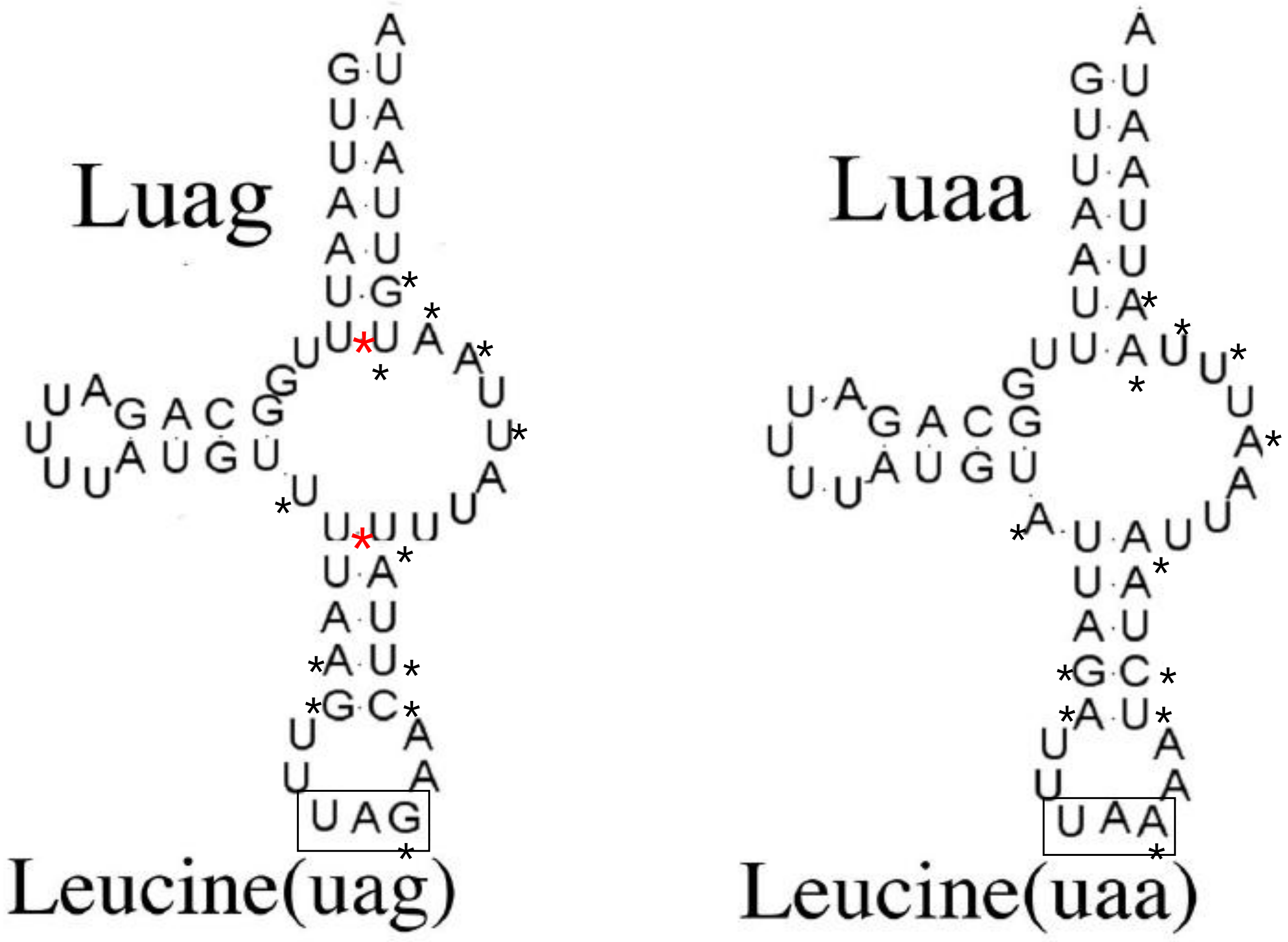

ClustalW Multiple alignment:

```
 Identities = 43/55 (78%), Gaps = 0

                ....|....| ....|....| ....|....| ....|....| ....|....| ....|
                        10          20        30         40         50
trnL-uag        GTTAATTTGG CAGATTTTAT GTTTTAAGTT TAGAACTTAT TTATTAATGT TAATA
                |||||||||| |||||||||| ||*|||**|| ||*||**||* |||*|****| |||||
trnL-uaa        GTTAATTTGG CAGATTTTAT GTATTAGATT TAAAATCTAA TTAATTTAAT TAATA
```

BLAST alignment:

```
 Length=55

 Score = 50.0 bits (54),  Expect = 2e-12
 Identities = 46/56 (82%), Gaps = 2/56 (4%)
 Strand=Plus/Plus
                ....|....| ....|....| ....|....| ....|....| ....|....| ....|
                        10          20        30         40         50
trnL-uag        GTTAATTTGG CAGATTTTAT GTTTTAAGTT TAGAA-CTTA TTTATTAATG TTAATA
                |||||||||| |||||||||| ||*|||**|| ||*||*||*| ||*|||*|** ||||||
trnL-uaa        GTTAATTTGG CAGATTTTAT GTATTAGATT TAAAATCTAA TTAATTTA-A TTAATA
```

Anticodon

Additional File 1. The comparison of gene sequences and secondary structures between *trnL1*-uag and *trnL2*-uaa.
