# Supplementary material for: The mitochondrial genome of Sinentomon erythranum (Arthropoda: Hexapoda: Protura): an example of highly divergent evolution
Source: BMC Evol Biol. 2011 Aug 27;11:246. doi: 10.1186/1471-2148-11-246 (PMC3176236; doi:10.1186/1471-2148-11-246)
Supplement: Additional File 2 — List of 24 mitochondrial genomes, which are not compatible with the "cox1-trnL2-cox2" pattern from Insecta and Crustacea, and not consistent with the "cox1-cox2" pattern from Chelicerata. [file 1471-2148-11-246-S2.PDF]

Additional File 2. List of 24 mitochondrial genomes, which are not compatible with the "cox1-trnL2-cox2" pattern from Insecta and Crustacea, and not consistent with the "cox1-cox2" pattern from Chelicerata.

Classfication	Species	Common names	GenBank Numbers	Rearrange type
**Insecta**				
Hymenoptera				
	Abispa ephippium	large potter wasp	NC_011520	4 copies of L2
	Diadegma semiclausum	wasp	NC_012708	Minor rearrangements
	Vanhornia eucnemidarum	parasitic wasp	NC_008323	Minor rearrangements
Phthiraptera				
	Bothriometopus macrocnemis	screamer louse	NC_009983	Reshuffled
	Campanulotes bidentatus compar	small pigeon louse	NC_007884	Reshuffled
	Heterodoxus macropus	wallaby louse	NC_002651	Reshuffled
Psocoptera				
	Lepidopsocid sp. RS-2001	scaly-winged barklouse	NC_004816	Major rearrangements
Thysanoptera				
	Thrips imaginis	plague thrips	NC_004371	Reshuffled
**Crustacea**				
Cephalocarida				
	Hutchinsoniella macracantha	cephalocarid crustacean	NC_005937	Minor rearrangements
Malacostraca				
Isopoda				
	Eophreatoicus sp. 14 FK-2009	freshwater isopod	NC_013976	Reshuffled
Decapoda				
	Geothelphusa dehaani	Japanese freshwater crab	NC_007379	Minor rearrangements
	Pagurus longicarpus	long-clawed hermit crab	NC_003058	Minor rearrangements
Maxillopoda				
Copepoda				
	Lepeophtheirus salmonis	salmon louse	NC_007215	Reshuffled
	Paracyclopina nana	cyclopoid copepod	NC_012455	Reshuffled

	Tigriopus californicus	tidepool copepod	NC_008831	Reshuffled
	Tigriopus japonicus	copepod	NC_003979	Reshuffled
Ostracoda				
Myodocopa				
	Vargula hilgendorfii	sea firefly	NC_005306	Major rearrangements
**Chelicerata**				
Acari				
Acariformes				
Eleutherengona				
	Panonychus citri	citrus red mite	NC_014347	Reshuffled
	Panonychus ulmi	European red spider mite	NC_012571	Reshuffled
	Tetranychus cinnabarinus	carmine spider mite	NC_014399	Reshuffled
	Tetranychus urticae	two-spotted spider mite	NC_010526	Reshuffled
Parasitengona				
	Ascoschoengastia sp. TATW-1	mite	NC_010596	Reshuffled
	Walchia hayashii	mite	NC_010595	Reshuffled
Parasitiformes				
	Varroa destructor	honeybee mite	NC_004454	Major rearrangements
