# Supplementary material for: The mitochondrial genome of Sinentomon erythranum (Arthropoda: Hexapoda: Protura): an example of highly divergent evolution
Source: BMC Evol Biol. 2011 Aug 27;11:246. doi: 10.1186/1471-2148-11-246 (PMC3176236; doi:10.1186/1471-2148-11-246)
Supplement: Additional File 3 — List of 24 taxa used in the phylogenetic analysis and the base composition of their mitochondrial genomes. [file 1471-2148-11-246-S3.PDF]

Additional File 3. List of 24 taxa used in the phylogenetic analysis and the base composition of their mitochondrial genomes.

Classification	Species / Common names	GenBank numbers	Genome length	AT%	AT-SKEW	GC-SKEW
**Onychophora**	*Opisthopatus cinctipes* /velvet worm	NC_014273	13673 nt	77.55%	-0.027	-0.012
**Chelicerata**						
Merostomata	*Limulus polyphemus* /Atlantic horseshoe crab	NC_003057	14985 nt	67.57%	0.111	-0.399
Arachnida	***Habronattus oregonensis*** **/spider**	**NC_005942***	**14381 nt**	**74.38%**	**-0.112**	**0.301**
Arachnida	***Centruroides limpidus*** **/Mexican scorpion**	**NC_006896***	**14519 nt**	**64.46%**	**-0.198**	**0.258**
Arachnida	*Eremobates cf. palpisetulosus* SEM-2008/sun spider	NC_010779	15083 nt	68.76%	0.091	-0.370
Arachnida	*Damon diadema* /whip scorion	NC_011293	14786 nt	63.19%	0.102	-0.473
**Myriapoda**						
Chilopoda	*Lithobius forficatus* /centipede	NC_002629	15695 nt	67.89%	0.087	-0.269
Diplopoda	*Narceus annularus* /millipede	NC_003343	14868 nt	63.74%	0.069	-0.397
**Crustacea**						
Remipedia	*Speleonectes tulumensis* /remipede	NC_005938	18372 nt	67.47%	0.247	-0.310
Cephalocarida	***Hutchinsoniella macracantha*** **/cephalocarid crustacean**	**NC_005937***	**16491 nt**	**71.62%**	**-0.193**	**0.313**
Branchiopoda	*Daphnia pulex* /common water flea	NC_000844	15333 nt	62.26%	0.011	-0.116
Branchiopoda	*Artemia franciscana* /brine shrimp	NC_001620	15822 nt	64.44%	-0.039	-0.005
Maxillopoda	*Pollicipes polymerus* /goose barnacle	NC_005936	15634 nt	67.04%	0.039	-0.134
Malacostraca	*Eriocheir sinensis* /Chinese mitten crab	NC_006992	16354 nt	71.63%	-0.015	-0.248
**Hexapoda**						
Protura	***Sinentomon erythranum*** **/proturan**	**HQ_199311***	**14491 nt**	**77.60%**	**-0.351**	**0.350**
Collembola	*Tetrodontophora bielanensis* /giant springtail	NC_002735	15455 nt	72.68%	0.045	-0.291
Collembola	*Gomphiocephalus hodgsoni* /springtail	NC_005438	15075 nt	74.08%	0.008	-0.175
Diplura	*Japyx solifugus* /two-pronged bristletail	NC_007214	15785 nt	64.82%	0.187	-0.290
Diplura	*Campodea fragilis* /dipluran	NC_008233	14965 nt	72.56%	0.062	-0.294
Insecta						
Archaeognatha	*Tricholepidion gertschi* /bristletail	NC_005437	15267 nt	68.60%	0.149	-0.239
Zygentoma	*Thermobia domestica* /firebrat	NC_006080	15152 nt	66.99%	0.067	-0.263
Lepidoptera	*Ostrinia furnacalis* /Asian corn borer	NC_003368	14536 nt	80.38%	0.032	-0.194
Coleoptera	*Tribolium castaneum* /red flour beetle	NC_003081	15881 nt	71.68%	0.109	-0.305
Diptera	*Anopheles gambiae* /African malaria mosquito	NC_002084	15363 nt	77.56%	0.032	-0.154

* with negative AT-skew and positive GC-skew
