# Supplementary material for: The mitochondrial genome of Sinentomon erythranum (Arthropoda: Hexapoda: Protura): an example of highly divergent evolution
Source: BMC Evol Biol. 2011 Aug 27;11:246. doi: 10.1186/1471-2148-11-246 (PMC3176236; doi:10.1186/1471-2148-11-246)
Supplement: Additional File 4 — AT-skew and GC-skew plot for 24 taxa used in phylogenetic analysis. [file 1471-2148-11-246-S4.PDF]

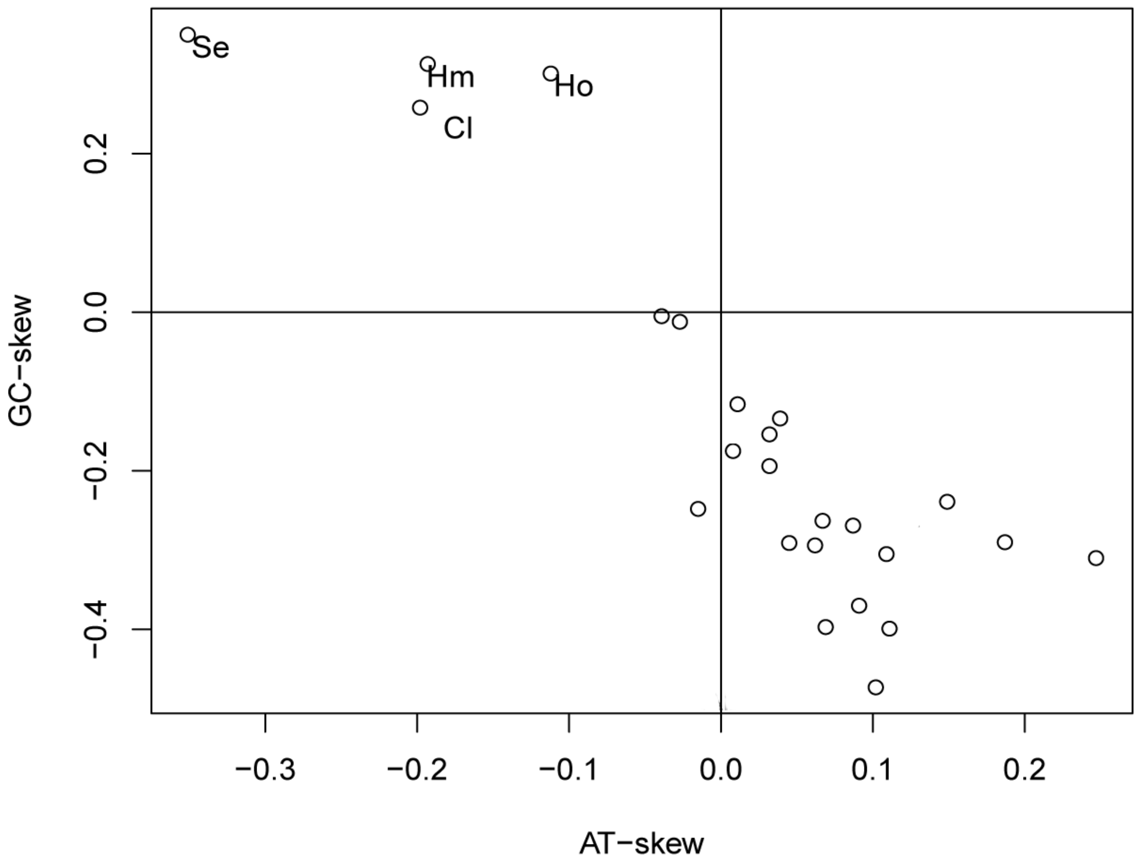

Additional File 4. AT-skew and GC-skew plot for 24 taxa used in the phylogenetic analysis.

Abbreviations: Se, *Sinentomon erythranum*; **Hm**, *Hutchinsoniella macracantha*; **Ho**, *Habronattus oregonensis*; **Cl**, *Centruroides limpidus.*
